# Could Camrelizumab Plus Chemotherapy Improve Clinical Outcomes in Advanced Malignancy? A Systematic Review and Network Meta-Analysis

**DOI:** 10.3389/fonc.2021.700165

**Published:** 2021-08-09

**Authors:** Chao Yang, Chang Xu, Xiang Li, Yaowen Zhang, Simeng Zhang, Tongyu Zhang, Yingshi Zhang

**Affiliations:** ^1^Department of Clinical Pharmacy, Shenyang Pharmaceutical University, Shenyang, China; ^2^Department of Ethnic Culture and Vocational Education, Liaoning National Normal College, Shenyang, China; ^3^The Chemical Laboratory, Liaoning Institute for Drug Control, Shenyang, China

**Keywords:** advanced malignancy, camrelizumab, efficacy, safety, network meta-analysis

## Abstract

**Purpose:**

Camrelizumab is a novel programmed cell death 1 (PD-1) inhibitor. To determine the efficacy and safety of the combination treatment of camrelizumab+chemotherapy and camrelizumab monotherapy, and determine which is the most suitable malignancy type to be treated with camrelizumab, we performed a systematic review and network meta-analysis.

**Methods:**

We searched PubMed, Embase, and the Cochrane Library for published clinical trials from database inception until April 2021. Studies that compared camrelizumab+chemotherapy and camrelizumab monotherapy in patients with advanced malignancy were included. We estimated odds ratios (ORs) with credible intervals (CIs) using network meta-analysis with random effects.

**Results:**

We included four clinical trials with 946 advanced malignancy patients. In terms of the efficacy evaluation of the objective response rate and progression-free survival, camrelizumab treatment for Hodgkin lymphoma (HL), camrelizumab treatment for esophageal squamous cell carcinoma (OSCC), and camrelizumab+chemo treatment for HL always ranked first. In terms of safety evaluation from leukocytopenia, hypothyroidism, and asthenia, camrelizumab treatment for OSCC and chemo always ranked first. This study was registered with PROSPERO, number CRD42021249193.

**Conclusions:**

Patients with advanced OSCC should be treated with camrelizumab. Patients with severely relapsed/refractory HL could use camrelizuma+chemo for combination treatment when they can tolerate adverse reactions.

**Systematic Review Registration:**

https://www.crd.york.ac.uk/prospero/display_record.php?RecordID=249193, PROSPERO (identifier, CRD42021249193).

## Introduction

Cancer is a major public health problem worldwide and is the second leading cause of death in the United States and the third leading cause of death in China. The overall cancer mortality rate has decreased by 31% due to the decrease in number of smokers, the improvement of medical standards, and the popularization of early screening (1). However, many patients still enter the advanced stage of malignancy due to failure of first-line treatment, metastasis, and recurrence. Moreover, for an advanced malignancy, the efficacy of traditional radiotherapy and chemotherapy is modest and cannot be used as a first-line rescue treatment strategy (2, 3).

Although the efficacy and safety of molecular targeted therapy and immunotherapy in a large number of clinical studies are inconsistent (4–7), it still cannot be used as the first-line treatment of advanced malignancy. At present, the recent emergence of programmed cell death 1 (PD-1)/programmed cell death ligand 1 (PD-L1) inhibitors may improve this situation (8–10). Camrelizumab (SHR-1210) is a humanized monoclonal antibody against PD-1 that shows efficacy and tolerance in many types of malignancies (11–13) and has received conditional approval in China for the treatment of patients with relapsed or refractory classical Hodgkin lymphoma who have received at least two previous systemic chemotherapies (14).

However, whether the combined application of camrelizumab and traditional chemotherapeutics could benefit more patients on the basis of tolerable adverse reactions has not yet been studied. The aim of our systematic review and network meta-analysis was to determine the efficacy and safety of the combination treatment of camrelizumab+chemotherapy and camrelizumab monotherapy, and to determine which is the most suitable malignancy type to be treated with camrelizumab.

## Materials and Methods

### Search Strategy and Selection Criteria

This research followed the Preferred Reporting Items for Systematic Reviews and Meta-Analyses (PRISMA) extension guidelines for network meta-analysis (15), and the project was prospectively registered on the PROSPERO database with registration number CRD42021249193 (16). We searched eligible trials in PubMed, Embase, and the Cochrane Library from inception until April 2021 with the terms “camrelizumab”, “SHR-1210”, “malignancy”, “cancer”, and their MeSH terms, and we restricted our results to randomized control trials (RCTs) or phase II/III clinical trials, with no restriction on language. We included publications comparing combination therapy of camrelizumab and chemotherapy (camrelizumab+chemo for short) with camrelizumab or chemotherapy alone, for patients with a primary diagnosis of advanced or refractory malignancy. Single-arm trials, trials with no combination therapy, and protocols were excluded.

### Data Extraction and Quality Assessment

Pairs of independent investigators (YC and XC) screened all titles, abstracts, and full texts after removing duplicates. Disputes and discrepancies were resolved by consensus and adjudication by an experienced investigator (ZYS). Details of the first author, publication year, study design, treatment arms, sample size, age, gender, efficacy outcomes reported, and safety outcomes reported were extracted from each included trial. The predefined efficacy outcomes were objective response rate (ORR), 6-month progression-free survival (6m-PFS), and 1-year progression-free survival (1y-PFS). Safety outcomes were leukocytopenia (all grade, 3–5 grade), hypothyroidism (all grade), and asthenia (all grade). We paid special attention to the problem of data duplication, and different publications with the same clinical trial number may cause the problem of data duplication. Data such as PFS that were not reported were measured on the graphs by manual measurement. We assessed the quality assessment of the included trials in accordance with the Cochrane Handbook for Systematic Reviews of Interventions (17).

### Data Synthesis and Analysis

The dichotomous outcome data from each network meta-analysis were summarized as odds ratios (ORs) and 95% credible intervals (CIs) using random-effects frequency network meta-analyses to increase the accuracy of the data (18). We used the surface under the cumulative ranking curve (SUCRA) to rank treatments for both efficacy and safety outcomes. The SUCRA score ranges from 0 to 1, and a higher SUCRA score indicates that there is a high possibility of becoming the optimal treatment (19). To provide an overview of the results from the network meta-analysis, we generated network diagrams and league charts from network forest plot rankings of efficacy and safety outcomes for combination therapy and monotherapy. Incoherence between indirect sources of evidence was statistically assessed using a global (design-by-treatment inconsistency model) and a local method (back calculation) (20, 21), and a network funnel plot was used to examine publication bias. All analyses were performed on StataSE version 15.1.

## Results

### General Characteristics of Included Trials

**Figure 1:**
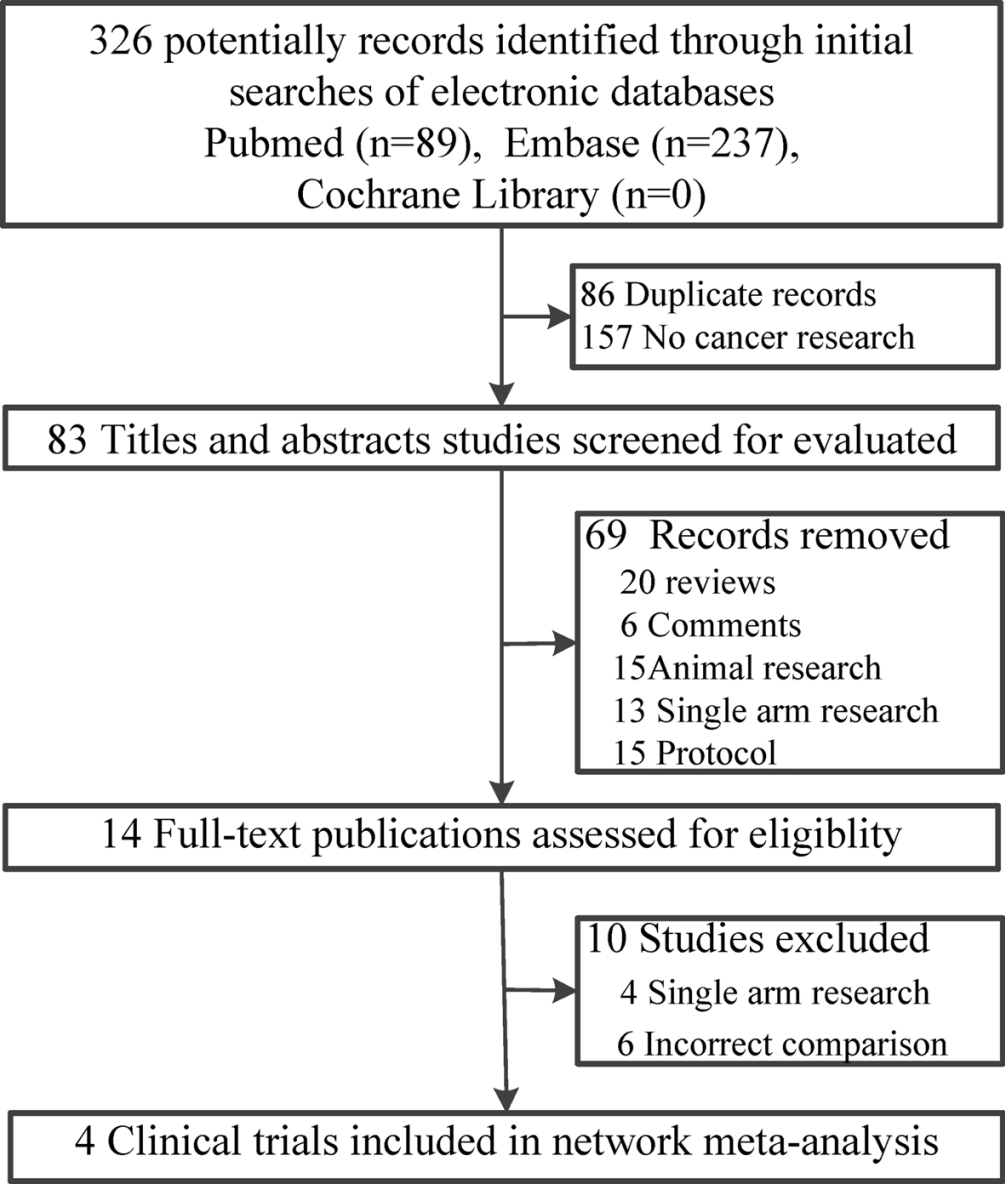
Flow diagram of including clinical trials.

Of the 326 records retrieved, 83 published studies met the inclusion criteria, and the full text was retrieved of 14 potentially eligible studies. After removing duplications, four clinical trials (22–25) including 946 patients with malignancy were published between 2019 and 2021 (**Figure 1**). Two of the included clinical trials were phase II studies (22, 25), and the other two were phase III studies (23, 24). One of the study patients had advanced non-squamous non-small-cell lung cancer (NSNLC) (23), and one had advanced or metastatic esophageal squamous cell carcinoma (OSCC) (24). The other two trials included patients with relapsed/refractory Hodgkin lymphoma (HL), and we paid special attention to the duplication of data published in these two studies (22, 25). For each included publication, the baseline information was basically homogeneous (**Table 1**). Even if all the included trials were open-label, the qualities were still suitable for network meta-analysis (**Figure S1**).

**Table 1:** Baseline Characteristic of included studies.

First author, year	Tumor types	Study design	Treatment arms	Sample size	Age	Gender	Efficacy outcomes reported	Safety outcomes reported
Liu et al. (22)	Relapsed/refractory Hodgkin lymphoma	Randomized phase II study	Camrelizumab	19	28 (18–44)	12/7	PFS, duration of response (DOR), ORR	Any grade, Grade 3–4
			Chemotherapy (Decitabine)+ camrelizumab	42	26 (12–66)	25/17		
Zhou et al. (23)	Advanced non-squamous non-small-cell lung cancer (CameL)	Randomized, open-label, multicenter, phase III trial	Camrelizumab+ Chemotherapy (carboplatin+ pemetrexed)	205	59 (54–64)	146/59	PFS, OS, ORR	Any grade, Grade 3–4
			Chemotherapy (carboplatin+ pemetrexed)	207	61 (53–65)	149/58		
Huang et al. (24)	Advanced or metastatic esophageal squamous cell carcinoma (ESCORT)	Multicenter, randomized, open-label, phase III study	Camrelizumab group	228	60 (54–65)	208/20	OS, PFS, DOR, ORR	Any grade, Grade 3–4
			Chemotherapy group	220	60 (54–65)	192/28		
Nie et al. (25)	Relapsed/refractory Classical Hodgkin Lymphoma	Two-arm, open-label, phase II study	Camrelizumab	19			ORR	Any grade, Grade 3–4
			Chemotherapy (Decitabine)+ camrelizumab	25				

### Efficacy of Combination Therapy and Monotherapy

**Figure 2:**
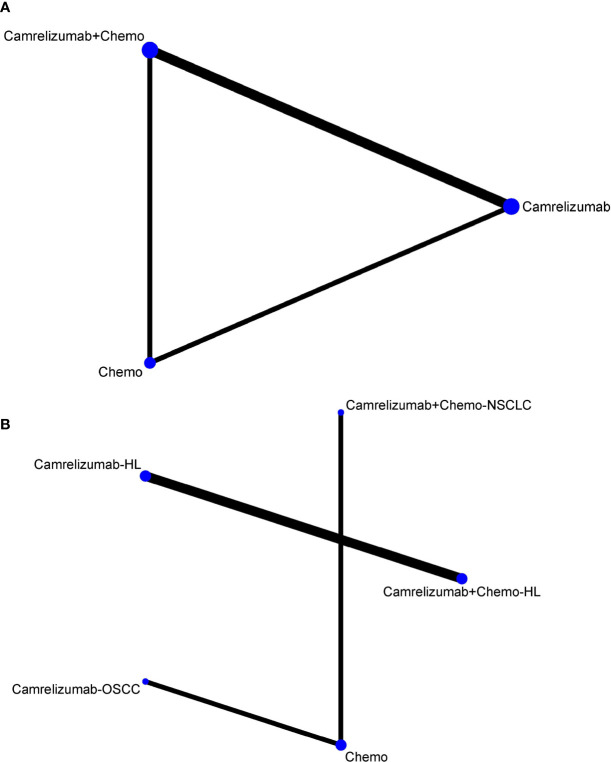
Network of eligible comparisons **(A)** without malignant subtypes, **(B)** with malignant subtypes. The width of the lines is proportional to the number of clinical trials comparing every pair of direct comparison, and the size of each node is proportional to the number of patients with malignant assigned. Chemo, chemotherapy; HL, Hodgkin lymphoma; OSCC, esophageal squamous cell carcinoma; NSCLC, non-squamous non-small-cell lung cancer.

**Figure 3:**
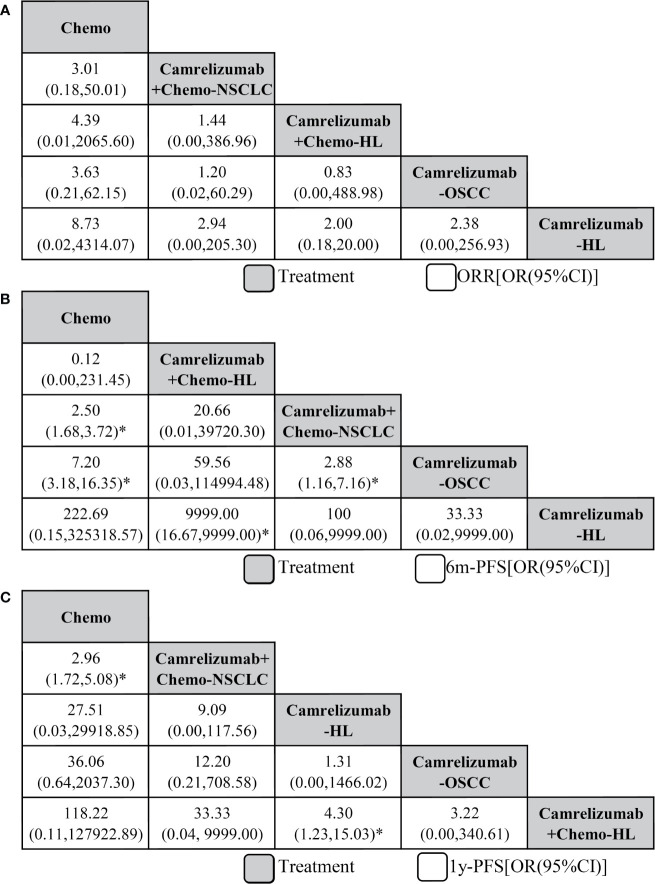
Network meta-analysis of efficacy outcomes including objective response rate **(A)**, 6-month progression-free survival **(B)**, and 1-year progression-free survival **(C)**. From right to left, treatments are ranked by mean rank and SUCRA score. Comparisons between treatments should be read from right to left, and the estimate is in the cell in common between the column-defining treatment and the row-defining treatment, and an OR less than 1 favors the row-defining treatment. To obtain OR for comparisons in the opposing direction, reciprocals should be taken. Chemo, chemotherapy; HL, Hodgkin lymphoma; OSCC, esophageal squamous cell carcinoma; NSCLC, non-squamous non-small-cell lung cancer. *Significant differences.

First, we analyzed the efficacy by ORR. Initially, we did not analyze the subtypes of malignant tumors, and network graphs are shown in **Figure 2A**. However, different types of malignant tumors may cause large sensitivity influences between combination therapy with camrelizumab+chemotherapy and monotherapy with camrelizumab. Therefore, we chose the malignant subtype for subsequent network meta-analysis, and the network plot is shown in **Figure 2B**. In terms of ORR efficacy, compared with chemotherapy (Chemo), camrelizumab monotherapy in Hodgkin lymphoma (camrelizumab-HL) ranked first based on the SUCRA score (OR=8.73, 95% CI: 0.02 to 4314.07), with no significant difference. The following treatment measures were camrelizumab monotherapy in esophageal squamous cell carcinoma (camrelizumab-OSCC), camrelizumab+chemo in HL, and camrelizumab+chemo in non-squamous non-small-cell lung cancer (NSCLC), respectively. There was no significant difference among all of the above comparison pairs (**Figure 3A**). Publication bias was low according to the netfunnel plot (**Figure S2**).

Second, we took 6m-PFS into consideration. Compared with Chemo, Camrelizumab for HL ranked first with no significant result, followed by Camrelizumab for OSCC with a significant difference (7.20, 3.18 to 16.35), Camrelizumab+Chemo for NSCLC with a significant difference (2.50, 1.68 to 3.72), and Camrelizumab+Chemo for HL. Moreover, a significant difference was also found in the comparison of the camrelizumab for OSCC *vs* camrelizumab+chemo for NSCLC groups (2.88, 1.16 to 7.16, **Figure 3B**). Third, in terms of 1y-PFS, Camrelizumab+Chemo for HL ranked first compared with Chemo, followed by Camrelizumab for OSCC, Camrelizumab for HL, and Camrelizumab+Chemo for NSCLC (2.96, 1.72 to 5.08). Significant differences were also found in the comparisons of camrelizumab+chemo for HL *vs* camrelizumab for HL (4.30, 1.23 to 15.03) and camrelizumab+chemo for NSCLC *vs* chemo (2.96, 1.72 to 5.08, **Figure 3C**).

Overall, we did not include overall survival (OS) as an indicator of efficacy because it was reported in only two studies. In terms of only considering the efficacy evaluation, the effects of camrelizumab alone and in combination with chemotherapy drugs are not much different, and the treatment effects are better in lymphoma and esophageal squamous cell carcinoma. For the completeness of the research, we also needed to conduct safety evaluations in the follow-up.

### Safety of Combination Therapy and Monotherapy

**Figure 4:**
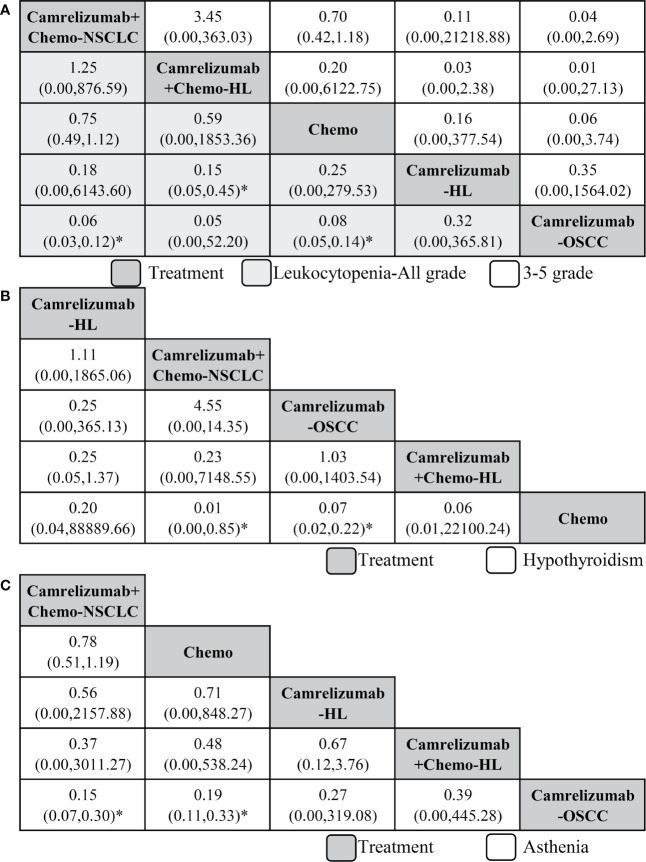
Network meta-analysis of safety outcomes including leukocytopenia **(A)**, hypothyroidism **(B)**, and asthenia **(C)**. From right to left, treatments are ranked by mean rank and SUCRA score. Comparisons between treatments should be read from right to left, and the estimate is in the cell in common between the column-defining treatment and the row-defining treatment, and an OR less than 1 favors the row-defining treatment. To obtain OR for comparisons in the opposing direction, reciprocals should be taken. Chemo, chemotherapy; HL, Hodgkin lymphoma; OSCC, esophageal squamous cell carcinoma; NSCLC, non-squamous non-small-cell lung cancer. *Significant differences.

First, we analyzed the safety of camrelizumab combination therapy and monotherapy by leukocytopenia. In terms of all-grade leukocytopenia, compared with camrelizumab+chemo for NSCLC, which ranked least, camrelizumab for OSCC ranked first with a significant difference (0.06, 0.03 to 0.12), followed by camrelizumab for HL, chemo and camrelizumab+chemo for HL. Significant results were also found in comparisons of camrelizumab for OSCC *vs* chemo (0.08, 0.05 to 0.14) and camrelizumab for HL *vs* camrelizumab+chemo for HL (0.15, 0.05 to 0.45). In terms of grade 3–5 leukocytopenia, the order of treatments was the same as that of all grades, and no significant results were found (**Figure 4A**).

In consideration of all-grade hypothyroidism, camrelizumab for HL ranked least, and compared with it, chemo ranked first, followed by camrelizumab+chemo for HL, camrelizumab for OSCC, and camrelizumab+chemo for NSCLC. Significant results were found in the comparisons of chemo *vs* camrelizumab for OSCC (0.07, 0.02 to 0.22) and chemo *vs* camrelizumab+chemo for NSCLC (0.01, 0.00 to 0.85, **Figure 4B**). Third, in terms of all-grade asthenia, camrelizumab+chemo for NSCLC also ranked least; compared with it, camrelizumab for OSCC ranked first (0.15, 0.07 to 0.30), followed by camrelizumab+chemo for HL, camrelizumab for HL and chemo. Significant differences were also found in the camrelizumab for OSCC *vs* chemo group (0.19, 0.11 to 0.33, **Figure 4C**).

In general, the treatment of camrelizumab could increase the risk of hypothyroidism. Moreover, the combination of camrelizumab and chemotherapy could increase the incidence of adverse reactions, especially in all grades of leukocytopenia and asthenia.

## Discussion

Our systematic review and meta-analysis followed PRISMA guidelines and was registered with PROSPERO collaboration, and we obtained the following results. First, we included four phase II/III clinical trials, which enrolled 946 patients with advanced malignancy (**Table 1** and **Figures 1**, **2**). Second, in the evaluation of efficacy from ORR and PFS, camrelizumab for HL, camrelizumab for OSCC, and camrelizumab+chemo for HL always ranked first. These results may suggest that camrelizumab has a good therapeutic effect in Hodgkin lymphoma and esophageal squamous cell carcinoma, which is independent to the combination with chemotherapy (**Figure 3**). Third, we performed a safety evaluation of camrelizumab, and we noticed that camrelizumab for OSCC and chemo always ranked first (**Figure 4**). The above results indicate that camrelizumab monotherapy is efficacious and safe in OSCC, while in HL, the combination of chemotherapy has little effect on efficacy and safety. Therefore, according to the results of our study, we suggest that patients with advanced OSCC should be treated with camrelizumab. For patients with HL, severely relapsed/refractory patients could use camrelizuma+chemo for combined treatment when they can tolerate adverse reactions.

The results we obtained could be verified in many published trials. In OSCC, Zhang B’s research determined that camrelizumab plus apatinib combined with liposomal paclitaxel and nedaplatin as first-line treatment demonstrated feasible antitumor activity and manageable safety in patients with advanced esophageal squamous cell carcinoma (26). Huang J’s study indicated that in the population of esophageal squamous cell carcinoma patients, SHR-1210 had a manageable safety profile and promising antitumor activity (27). Yan Z showed that a PD-1 inhibitor combined with an antiangiogenic agent is effective and safe for the treatment of esophageal squamous cell carcinoma, and camrelizumab is worth investigating in clinical trials (28). When considering HL, camrelizumab demonstrated a high response rate, durable response, and controllable safety in Chinese patients with relapsed or refractory classical Hodgkin lymphoma, and PD-1 is a well-recognized attractive target. This multicenter, single-a study demonstrates a new safe and effective treatment option in this setting (12). Liu Y found that decitabine plus camrelizumab resulted in longer PFS than camrelizumab alone in patients with relapsed/refractory classical Hodgkin lymphoma, which is very similar to our result (22). Therefore, camrelizumab monotherapy or in combination may be a new strategy for the treatment of HL (29).

In terms of NSCLC, we found that the efficacy and safety of camrelizumab were not as good as those in HL and OSCC. However, other published studies have shown that combined apatinib and camrelizumab showed encouraging antitumor activity and acceptable toxicity in chemotherapy-pretreated patients with advanced non-squamous NSCLC. Patients with STK11/KEAP1 mutations might derive more benefits from this combination (30). Moreover, camrelizumab administration combined with microwave ablation was safe in the treatment of advanced NSCLC, and the combination improved the ORR of camrelizumab alone compared to previous reports (31). The reason for the inconsistency may be that the sample size is not large enough, or it is not combined with locoregional therapy, which needs to be confirmed by large-scale sample clinical trials published in the future.

Regarding the safety of camrelizumab, our research and other publications have proven that camrelizumab is well tolerated, but we found that treatment with camrelizumab has a risk of all-grade hypothyroidism (**Figure 4B**). Because camrelizumab is a new agent, there is no specific analysis of the causes and mechanisms of hypothyroidism. After searching the literature, we found that camrelizumab has been reported to have this risk, and we wait for the explanation of mechanism and confirmation of follow-up basic research (13, 31, 32).

There are also some limitations in our research. First, due to fewer original studies and fewer articles included, large-scale sample clinical trials will be published in the future. Second, due to the small number of included publications and the scattered subtypes of malignant tumors, we could not perform pairwise meta-analysis. Third, due to fewer articles included, some arms in the network meta-analysis have only one study, and there are fewer loops in the networkplot, so we cannot perform consistency analysis.

In conclusion, our results suggest that patients with advanced OSCC are recommended to take camrelizumab for treatment. In patients with relapsed/refractory HL, camrelizumab monotherapy and in combination with chemotherapy is effective and safe. Camrelizumab can be used as a first-line rescue treatment strategy. However, this conclusion still needs to be confirmed in large-scale, randomized double-blind controlled trials.

## Data Availability Statement

The original contributions presented in the study are included in the article/**Supplementary Material**. Further inquiries can be directed to the corresponding author.

## Author Contributions

CY: concept, design, statistics, data collection, manuscript writing, final approval. CX: design, statistics, data collection. XL: concept, data collection. YWZ: statistics, manuscript writing. SZ: statistics, data collection. TZ: statistics, data collection. YSZ: concept, design, statistics, data collection, manuscript writing, final approval.

## Conflict of Interest

The authors declare that the research was conducted in the absence of any commercial or financial relationships that could be construed as a potential conflict of interest.

## Publisher’s Note

All claims expressed in this article are solely those of the authors and do not necessarily represent those of their affiliated organizations, or those of the publisher, the editors and the reviewers. Any product that may be evaluated in this article, or claim that may be made by its manufacturer, is not guaranteed or endorsed by the publisher.

## Supplementary Material

Supplementary Figure 1 Risk of bias summary.

Supplementary Figure 2 Netfunnel of objective response rate.
