# Supplementary figures and images for: Could Camrelizumab Plus Chemotherapy Improve Clinical Outcomes in Advanced Malignancy? A Systematic Review and Network Meta-Analysis
Source: Front Oncol. 2021 Aug 9;11:700165. doi: 10.3389/fonc.2021.700165 (PMC8415159; doi:10.3389/fonc.2021.700165)

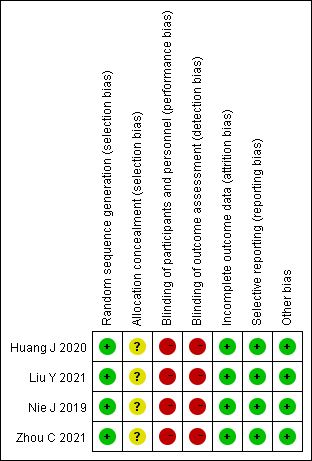

Supplement: Supplementary Figure 1 — Risk of bias summary. [file Image_1.png]

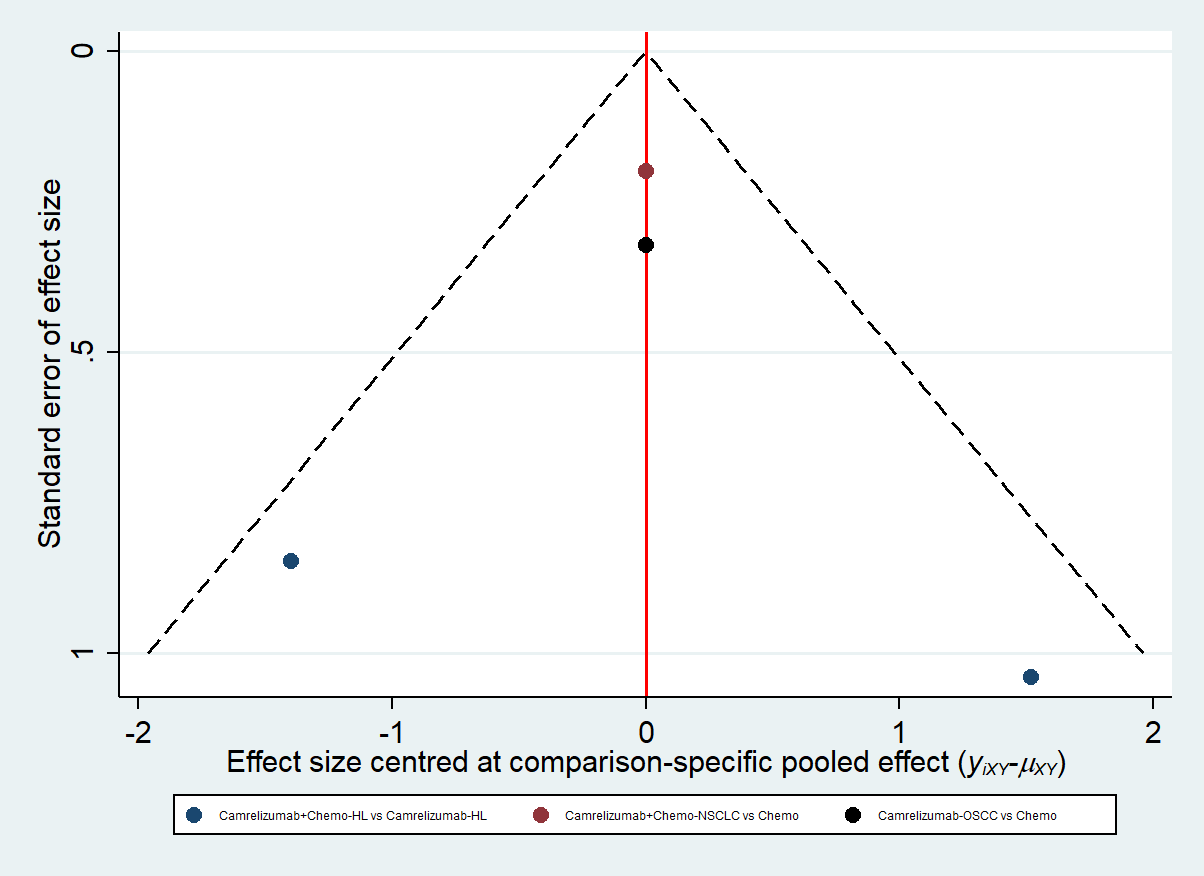

Supplement: Supplementary Figure 2 — Netfunnel of objective response rate. [file Image_2.png]
